# Skin symptoms in bakery and auto body shop workers: associations with exposure and respiratory symptoms

**DOI:** 10.1007/s00420-012-0760-x

**Published:** 2012-03-13

**Authors:** Victoria Arrandale, Tim Meijster, Anjoeka Pronk, Gert Doekes, Carrie A. Redlich, D. Linn Holness, Dick Heederik

**Affiliations:** 1 Centre for Research Expertise in Occupational Disease, University of Toronto, 223 College St, Toronto, ON M5T 1R4 Canada; 2 TNO Quality and Safety, Zeist, The Netherlands; 3 Environmental Epidemiology Department, Institute for Risk Assessment Sciences, Utrecht University, Utrecht, The Netherlands; 4 Yale University School of Medicine, New Haven, CT USA

**Keywords:** Occupational exposure, Skin symptoms, Respiratory symptoms, Exposure–response relationships

## Abstract

**Purpose:**

Despite the importance of skin exposure, studies of skin symptoms in relation to exposure and respiratory symptoms are rare. The goals of this study were to describe exposure–response relationships for skin symptoms, and to investigate associations between skin and respiratory symptoms in bakery and auto body shop workers.

**Methods:**

Data from previous studies of bakery and auto body shop workers were analyzed. Average exposure estimates for wheat allergen and isocyanates were used. Generalized linear models were constructed to describe the relationships between exposure and skin symptoms, as well as between skin and respiratory symptoms.

**Results:**

Data from 723 bakery and 473 auto body shop workers were analyzed. In total, 5.3 % of bakery and 6.1 % of auto body shop workers were female; subjects’ mean age was 39 and 38 years, respectively. Exposure–response relationships were observed in auto body shop workers for itchy or dry skin (PR 1.55, 95 % CI 1.2–2.0) and work-related itchy skin (PR 1.97, 95 % CI 1.2–3.3). A possible exposure–response relationship for work-related itchy skin in bakery workers did not reach statistical significance. In both groups, reporting skin symptoms was strongly and significantly associated with reporting respiratory symptoms, both work-related and non-work-related.

**Conclusions:**

Exposure–response relationships were observed for skin symptoms in auto body shop workers. The lack of significant exposure–response associations in bakery workers should be interpreted cautiously. Workers who reported skin symptoms were up to four times more likely to report respiratory symptoms. Improved awareness of both skin and respiratory outcomes in exposed workers is needed.

**Electronic supplementary material:**

The online version of this article (doi:10.1007/s00420-012-0760-x) contains supplementary material, which is available to authorized users.

## Introduction

The connection between skin and respiratory systems in occupational disease is a growing area of research interest (Redlich and Herrick 2008). Specifically, there is interest in determining whether the skin can be an important route of sensitization for occupational allergens and subsequent development of occupational respiratory symptoms, including asthma. Research in this area is challenging, in part due to the organ system silos that have historically existed in medicine and epidemiological research.

Recent evidence from animal models suggests that after sensitization through skin exposure to some high (e.g., latex) and low (e.g., trimellitic anhydride, toluene diisocyanate (TDI)) molecular weight agents, an asthma-like response can be elicited upon inhalation exposure (Vanoirbeek et al. 2004; Zhang et al. 2009). Evidence of possible cross-system sensitization and elicitation in humans is scarce. Among methylene diphenyl diisocyanate (MDI)-exposed workers, Petsonk et al. (2000) observed that subjects reporting skin staining (a proxy for skin exposure) were more likely to report asthma-like symptoms.

Despite the possibility that skin exposures can contribute to the burden of respiratory disease, studies focussing on skin exposure, and specifically on exposure–response studies for skin symptoms and/or sensitization, are rare. This lack of evidence limits the ability to infer causality between skin exposure and response, and may ultimately hamper efforts to better control both skin exposure as well as skin and respiratory symptoms in the workplace. Studies on skin symptoms in relation to exposure do exist (de Joode et al. 2007; Sripaiboonkij et al. 2009a, b), but even less information is available on the associations between exposure, skin, and respiratory symptoms as well as the relationship between skin and respiratory effects. Many occupational studies report the prevalence of both skin and respiratory symptoms but rarely explore the relationship between the two, or the prevalence of these symptoms coexisting. Lynde et al. (2009) reported that among male cleaners, those with skin symptoms were more likely to report respiratory symptoms.

The mechanisms of airborne and skin exposure are complex. Airborne and skin exposures can be related if they share sources, but these associations are so far poorly studied (Schneider et al. 1999). Associations between skin and airborne exposures have been reported for bitumen and pyrene in road pavers, 1,6-hexamethylene diisocyanate (HDI) in spray painters, methylene bisphenyl isocyanate (MDI) in foundry works, solvents in spray painters, and nickel exposure in primary industries (McClean et al. 2004; Burstyn et al. 2002; Chang et al. 2007; Fent et al. 2008; Liljelind et al. 2010; Hughson and Cherrie. 2005). In two other studies, both involving pesticide exposure, there was no association found between skin and airborne exposure. The authors attribute this lack of association to the fact that the primary source of skin exposure was likely contact with contaminated foliage rather than the settling of airborne pesticide (Flack et al. 2008; Aprea et al. 2009).

Bakery and auto body shop workers have both skin and respiratory exposures to known occupational allergens, making them good candidates for further study of exposure–response relationships for skin symptoms, as well as the relationship between skin and respiratory symptoms. Bakery and auto body shop workers are at increased risk of occupational asthma (OA) as well as occupational skin disease (OSD) due to their workplace exposures: flour dust and diisocyanates, respectively (McDonald et al. 2005, 2006). Flour dust is a common cause of occupational asthma in bakers. Flour dust, which includes wheat and α-amylase allergens among others, contains high molecular weight (HMW) antigens which act through an IgE-mediated (Type I) immunological pathway to cause OA and contact urticaria, and can also cause contact dermatitis through a Type IV (cell-mediated) mechanism (Nethercott and Holness 1989). Isocyanates are a heterogeneous group of compounds, including monomers and oligomers, categorized as low molecular weight (LMW) antigens. The mechanism of action leading to isocyanate-induced OA is not yet fully understood and though IgE (Type I)-mediated processes do appear to play a role in some cases, other unrevealed mechanisms play a role in respiratory sensitization (Maestrelli et al. 2009; Wisnewski 2007). Similar to flour dust, isocyanates can also cause contact dermatitis (Type IV) (Donovan et al. 2009; Frick et al. 2003).

The goals of this study are to describe the exposure–response relationships for skin symptoms in both bakery workers and auto body shop workers, and to investigate the association between skin and respiratory symptoms in these two groups.

## Methods

Reports on respiratory outcomes in both the bakery and auto body shop workers studies have been published previously (Pronk et al. 2007; Jacobs et al. 2008). Workers were asked to complete a questionnaire on respiratory and skin symptoms, an exposure questionnaire, and also to provide a blood sample for analysis. For this analysis, subjects were required to have complete data for both respiratory and skin symptoms, as well as atopy and workplace allergen-specific IgE. In total, 723 bakery workers and 472 auto body shop workers were included in this analysis, which is a slightly different study population than previous publications (Pronk et al. 2007; Jacobs et al. 2008).

### Exposure

In both groups (bakery and auto body shop workers), exposure was estimated based on existing data sets of personal airborne exposure measurements (Pronk et al. 2006a; Meijster et al. 2007). Cumulative monthly hexamethylene diisocyanate (HDI) exposure was estimated using task-based measurements of airborne diisocyanates combined with self-reported monthly frequencies of task completion as was described previously (Pronk et al. 2007). This exposure metric was then divided by the self-reported average number of hours worked per month to determine the long-term average isocyanate exposure of these workers (μg-NCO*m^−3^) that facilitated comparison with the bakery workers. Average wheat exposure for bakery workers was estimated using subjects’ work characteristics (exposure determinants) reported on the questionnaire combined with an exposure model constructed by Meijster et al. (2007), to predict average wheat exposures (μg-dust*m^−3^) for each subject.

A relatively small number of task-based skin exposure measurements were available for isocyanate exposure in auto body shops, but no comparable exposure measurements were available in bakery workers. As a result, this study investigates the exposure–response relationships for skin symptoms, using airborne exposure as a proxy for skin exposure in both working populations. In auto body shop workers, airborne exposure was not significantly associated with having a detectable skin exposure sample (OR 1.34, 0.97–1.84), but the analysis was limited by small number of samples and a direct correlation was not calculated (Pronk et al. 2006b).

### Specific IgE and atopy

Specific IgE was measured using commercially available kits as previously described (Pronk et al. 2007; Jacobs et al. 2008). In bakery workers, specific IgE was measured for wheat protein (Bakery, Pharmacia, Unicap System, Pharmacia Diagnostics, Uppsala, Sweden); in auto body shop workers, specific IgE to HDI oligomers (N100-HAS) was measured (Isocyanates: Phadia, Uppsala, Sweden). All samples were also tested for specific IgE to common aeroallergens (house dust mite, cat, dog, grass, or birch pollen) (Doekes et al. 1996). Analytical results were dichotomized and IgE (work-related or common allergens) was considered elevated if above 0.35 kU/L. Subjects were classified atopic if they had elevated IgE in response to at least one of the common aeroallergens.

### Symptoms

Respiratory symptoms and skin symptoms were reported on a self-completed questionnaire derived from the International Union Against Tuberculosis and Lung Disease (IUATLD) and the Medical Research Council—European Community of Coal and Steel (MRC-ECCS) for the bakery workers, and from the British Medical Research Council (BMRC) respiratory questionnaire for auto body shop workers (Burney et al. 1989; van der Lende and Orie 1972; Medical Research Council on the Aetiology of Chronic Bronchitis 1960). Information on cough, phlegm, wheeze, chest tightness, shortness of breath, and self-reported asthma was included. A variable describing asthma-like symptoms (wheezing, chest tightness, current/previous asthma) was constructed using the individual symptom responses. Skin itch and dry skin were reported on the questionnaire; a dichotomous variable describing the presence of either itchy or dry skin was constructed. Work-related symptoms were explicit items on the questionnaire. Subjects were asked directly whether they have itchy skin at work and whether they experience asthma-like symptoms at work. No work-related symptom variables were constructed post hoc.

### Additional variables

Age, sex, smoking (current and historical) as well as years working were self-reported on the questionnaire.

### Analyses

Iterative non-parametric regression models (smoothing splines) with generalized additive models (PROC GAM) were first used to explore the shape of the exposure–response relationships for skin outcomes at the population level. These models were used to explore unadjusted non-linear relationships between estimated exposure and symptoms outcomes. Generalized cross-validation (GCV) was used to select the smoothing parameter degrees of freedom (*df*); the *df* selected were limited to four to avoid large fluctuations that are likely not biologically relevant (Hastie 1990).

Generalized linear models (SAS PROC GENMOD) with a log function were used to estimate unadjusted and adjusted prevalence ratios (PR) for the associations between exposure, atopy, specific sensitization, and symptoms. Adjusted models included atopy, work-related specific IgE sensitization, age, and sex; respiratory symptom models were additionally adjusted for smoking status. Sensitivity analyses were completed to explore whether atopy and specific sensitization were modifying the exposure–response relationships. Exposure–response relationships were investigated in models where atopic and specific sensitized subjects were excluded. All PR estimates for exposure effects are reported as the PR associated with an inter-quartile range (IQR) increase in exposure.

Additionally, relationships between skin and respiratory symptoms were explored using generalized linear models (PROC GENMOD) as described above with the same covariates and including sensitivity analyses to explore the effect of atopy and work-related specific sensitization. All analyses were completed in SAS v.9 software (SAS Institute Inc., Cary, NC, USA).

## Results

Both the auto body shop and bakery workers were predominantly male with an average age of approximately 38 and 39 years, respectively (Table 1). The distribution of smoking status was similar between the two groups, though there were more never-smokers among the bakery workers.

**Table 1:** Demographics and symptom frequencies for both auto body repair and bakery workers

	Auto body repair workers	Bakery workers
Demographics		
Overall, *n*	473	723
Female, *n* (%)	29 (6.1)	38 (5.3)
Age, mean (sd)	38.0 (11)	39.0 (11)
Current smoker, *n* (%)	173 (37)	238 (33)
Former smoker, *n* (%)	130 (28)	157 (22)
Never smoker, *n* (%)	170 (36)	328 (45)
Years working, mean (sd)	17.6 (11)	14.4 (11)
Symptoms, *n* (%)		
Cough	65 (14)	83 (12)
Wheeze, ever	111 (24)	111 (15)
Asthma, ever	72 (15)	71 (9.8)
Asthma symptoms	134 (28)	174 (24)
Work-related asthma symptoms	20 (4.2)	15 (2.1)
Dry skin in the last 12 months	113 (24)	188 (26)
Itchy skin in the last 12 months	50 (11)	208 (29)
Either itchy or dry skin in the last 12 months	134 (28)	265 (37)
Work-related itchy skin	40 (8.5)	122 (17)
Atopy and specific IgE, *n* (%)		
Atopy	169 (36)	245 (34)
HDI-specific IgE	10 (2.1)	
Wheat-specific IgE		82 (11)

The prevalence of atopy among bakery and auto body shop workers was similar (34 vs. 36 %, respectively) but the prevalence of specific sensitization to workplace allergens was higher among bakery workers (Table 1). Eleven percent of bakery workers had wheat-specific IgE; only 2 % of auto body shop workers had HDI-specific IgE.

Differences between the bakery and auto body shop workers were observed in symptom frequencies (Table 1). We observed slightly more respiratory symptoms in auto body shop workers and more skin symptoms in bakery workers. Estimated average exposure among auto body repair shop workers ranged from 0 to 353 μg-NCO*m^−3^ (IQR 21.4), and among bakery workers from 0.35 to 95.6 μg-wheat*m^−3^ (IQR 32.9) based on the previously collected exposure measures.

**Fig. 1:**
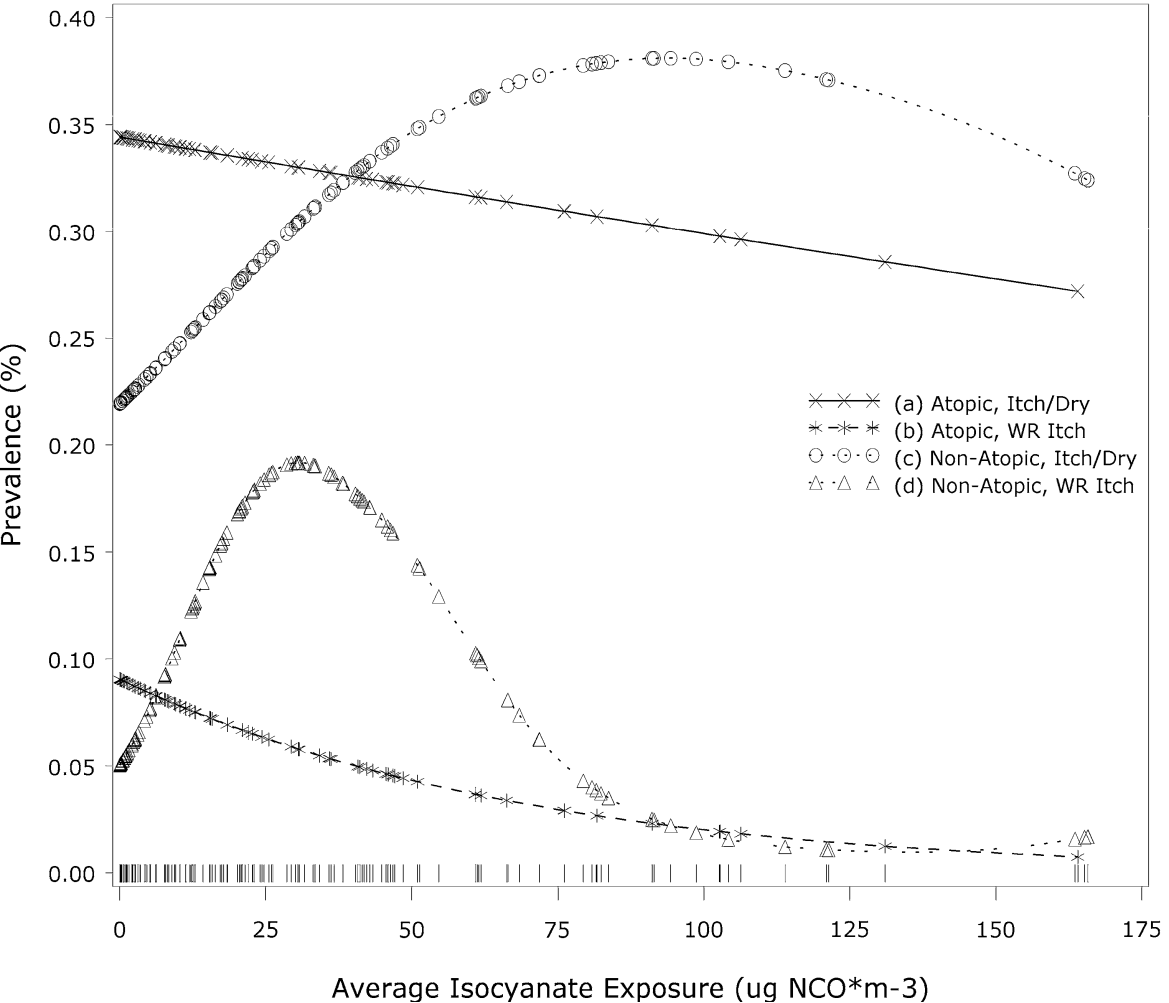
Auto body shop workers: associations between average isocyanate exposure and skin symptoms, shown in smoothed plots, stratified by atopy. Data rug indicates the distribution of observations by exposure level. *a* Itchy or dry skin in atopic subjects (linear: NS; spline: NS), *b* work-related itchy skin in atopic subjects (linear: NS; spline: NS), *c* itchy or dry skin in non-atopic subjects (linear: NS; spline: *df* = 1.05, *p* < 0.05), *d* work-related itchy skin in non-atopic subjects (linear: NS; spline: *df* = 3.71, *p* < 0.05)

**Fig. 2:**
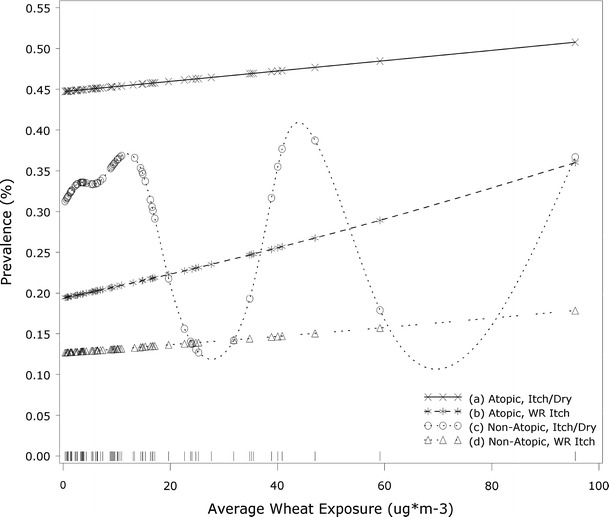
Bakery workers: Associations between average wheat exposure and skin symptoms, shown in smoothed plots, stratified by atopy. Data rug indicates the distribution of observations by exposure level. *a* Itchy or dry skin in atopic subjects (linear: NS; spline: NS), *b* work-related itchy skin in atopic subjects (linear: NS; spline: NS), *c* itchy or dry skin in non-atopic subjects (linear: NS; spline: NS), *d* work-related itchy skin in non-atopic subjects (linear: NS; spline: NS), atopic subjects (linear: NS; spline: NS)

Smoothing splines (Figs. 1, 2) show the shape of the exposure–response distribution for skin symptoms at a population level, stratified by atopy. Among bakers, the exposure–response relationship for skin symptoms appears to be linear in both the atopic and non-atopic groups. However, in auto body shop workers, a bell-shaped distribution is supported (*df* = 3.7; *p* < 0.05) in non-atopic subjects. Similar analyses for respiratory symptoms have been previously reported for both the bakery and auto body shop workers (Pronk et al. 2007; Jacobs et al. 2008). Graphs for respiratory symptom models directly comparable to the skin symptom models presented here are provided for comparison in the Supplemental Material.

In auto body shop workers (Table 2), statistically significant exposure–response relationships were observed for itchy or dry skin (PR 1.56, 95 % CI 1.2–2.0) and work-related itchy skin (PR 1.97, 95 % CI 1.2–3.3); a similar trend was observed in the bakery workers for work-related skin symptoms but this did not reach significance (Table 2).

**Table 2:** Results of generalized linear models describing the simple relationship between exposure, symptoms, atopy, and specific IgE

Independent variable	Dependant variable	PR (95 % CI)
Auto body repair workers (*n* = 473)		
Average isocyanate exposure (μg-NCO*m^−3^)	Itchy or dry skin	1.56 (1.2–2.0)
	WR itchy skin	1.97 (1.2–3.3)
	Atopy	0.83 (0.7–1.0)
	HDI-specific IgE	10.0 (1.4–73)
Atopy	Itchy or dry skin	1.26 (1.0–1.7)
	WR itchy skin	0.80 (0.4–1.5)
HDI-specific IgE	Itchy or dry skin	1.86 (1.1–3.2)
	WR itchy skin	1.03 (0.2–6.8)
Bakery workers (*n* = 723)		
Average wheat exposure (μg*m^−3^)	Itchy or dry skin	0.96 (0.8–1.1)
	WR itchy skin	1.16 (0.9–1.5)
	Atopy	0.91 (0.8–1.1)
	Wheat-specific IgE	1.12 (0.8–1.5)
Atopy	Itchy or dry skin	1.45 (1.2–1.8)
	WR itchy skin	1.67 (1.5–3.1)
Wheat-specific IgE	Itchy or dry skin	1.22 (0.9–1.6)
	WR itchy skin	2.17 (1.5–3.1)

Each reported prevalence ratio (PR) was estimated from a separate model. Models adjusted for age and sex. (*WR* work-related)

In auto body shop workers (Table 2), exposure was significantly related to specific HDI sensitization (PR 10.0, 95 % CI 1.4–73), with wide confidence limits likely due to the small number of sensitized subjects. HDI-specific sensitization was associated with itchy or dry skin (PR 1.86, 95 % CI 1.1–3.2) but not work-related itchy skin. Atopy predicted itchy or dry skin in auto body shop workers (PR 1.26, 95 % CI 1.0–1.7) but not work-related itchy skin.

Among bakery workers (Table 2), wheat exposure was not related to having wheat-specific sensitization, but wheat-specific sensitization was associated with work-related itchy skin (PR 2.17, 95 % CI 1.5–3.1). Atopy was associated with both itchy or dry skin (PR 1.45, 95 % CI 1.2–1.8) and work-related itchy skin (PR 1.67, 95 % CI 1.2–2.3).

In both groups, exposure was negatively associated with atopy, though this relationship only reached significance in the auto body shop workers (Table 2).

When atopy and specific sensitization were added to exposure–response models for skin symptoms, the effect on prevalence ratios due to exposure remained relatively unchanged in both groups (Table 3). Removing the atopic and sensitized (work-related specific IgE) subjects also did not change the exposure relative risk estimates (results not shown).

**Table 3:** Prevalence ratio (PR) of symptoms per interquartile range (IQR) increase in average exposure

Outcome	Covariates	PR (95 % CI)
Bakery workers (*n* = 723)		
Either itchy or dry skin in last 12 months	A, S, Atp, IgE	0.96 (0.8–1.1)
Work-related itchy skin	A, S, Atp, IgE	1.14 (0.9–1.5)
Auto body repair workers (*n* = 473)		
Either itchy or dry skin in last 12 months	A, S, Atp, IgE	1.55 (1.2–2.0)
Work-related itchy skin	A, Atp, IgE	1.97 (1.2–3.3)

Models adjusted for atopy and specific sensitization in addition to age, sex, and smoking as described

*A* age, *S* sex, *Sm* smoking, *Atp* atopy, *IgE* work-related specific IgE

The association between reporting skin symptoms and reporting respiratory symptoms was investigated separately (Table 4). In both auto body shop and bakery workers, reporting itchy/dry skin and work-related itchy skin was significantly associated with reporting wheeze and asthma-like symptoms. Both work-related and non-work-related skin symptoms were significantly associated with work-related chest tightness in auto body shop workers. In bakery workers, work-related itchy skin was not significantly associated with work-related chest tightness.

**Table 4:** Association between skin symptoms and respiratory symptoms in both bakery and auto body repair workers

Predictor	Outcome	Auto body repair workers	Bakery workers
		PR (95 % CI)	PR (95 % CI)
Itchy or dry skin in last 12 months	Wheeze, ever	2.01 (1.5–2.8)	1.94 (1.4–2.7)
	Asthma-like symptoms	1.83 (1.4–2.4)	1.78 (1.4–2.3)
	WR asthma symptoms	4.06 (1.6–10.1)	3.90 (1.2–12.2)
Work-related itchy skin	Wheeze, ever	2.50 (1.7–3.6)	1.60 (1.1–2.3)
	Asthma-like symptoms	2.12 (1.5–3.0)	1.54 (1.2–2.0)
	WR asthma symptoms	3.61 (1.4–9.4)	2.15 (0.7–6.3)

Reported as prevalence ratio of respiratory symptoms, adjusted for age, sex, smoking, and atopy with 95 % CI

## Discussion

Significant exposure–response relationships were observed between estimated exposure to diisocyanates (μg-NCO*m^−3^) and skin symptoms in auto body shop workers. Such associations have not been previously reported. Though similar trends were observed between wheat exposure and work-related skin symptoms in bakery workers, the associations did not reach statistical significance.

Both auto body repair and bakery workers who reported skin symptoms were consistently and significantly more likely to report work-related and non-work-related respiratory symptoms. These findings are comparable with results of Lynde et al. (2009) who showed that male cleaners with a skin rash were more likely to report respiratory symptoms, particularly work-related respiratory symptoms.

The prevalence of skin symptoms reported in auto body shop workers and bakery workers is similar to previous studies of skin outcomes in these populations. Randolph et al. (1997) reported that 32 % of HDI-exposed spray painters reported hand dermatitis, while Daftarian et al. (2002) found 35 % of TDI-exposed workers to have skin symptoms. Cullinan et al. (2001) found that 11 % of bakery and flour mill workers had skin symptoms. Steiner et al. (2011) reported that 19 % of all bakers and 31 % of high-risk (higher likelihood of exposure) bakers reported at least one skin symptom in the last 12 months.

Previous research supports that self-reported skin symptoms are predictive of skin disease. However, some results suggest that self-reported skin symptoms may overestimate (Smit et al. 1992; Lynde et al. 2009) or underestimate (Holness et al. 1995) the prevalence of disease when compared with a physician examination. The use of picture-based questionnaires and self-reported doctor-diagnosed dermatitis may provide a prevalence estimate closer to that of physician diagnoses, but may also underestimate prevalence (Smit et al. 1992).

Skin symptoms may be due to irritant or different immunologic (Type I or Type IV) mechanisms. Though it is possible to differentiate between these outcomes in the clinical setting, it is not possible to differentiate using symptoms reported on the questionnaire alone. The strong relationship between wheat-specific IgE and work-related itchy skin supports a role for the IgE-mediated (Type I) allergy in the development of work-related skin symptoms in bakery workers. Parallel results for respiratory symptoms (Supplemental Material) also demonstrate strong relationships between wheat-specific IgE and both asthma-like symptoms and work-related chest tightness. It is not possible to model the potential role of Type IV allergy or irritant mechanisms in symptom development in this study.

The bell-shaped (non-linear) distribution observed for non-atopic auto body shop workers in the smoothing splines (Fig. 1) may be the result of a healthy worker effect, with fewer symptomatic subjects at the higher exposure levels. A healthy worker effect was also suggested by the negative association between exposure and atopy in both the auto body shop and bakery workers (Table 2).

The prevalence of work-related allergen-specific sensitization was five times higher in bakery workers (11 %) compared to auto body shop workers (2 %). The low prevalence of HDI-specific IgE sensitization is well documented in other studies and is commonly interpreted as indicating mechanisms other than IgE sensitization are responsible for the development of symptoms in exposed workers (Maestrelli et al. 2009; Wisnewski. 2007).

Atopy and work-related sensitization were strongly associated in both auto body shop workers (PR 13.8, 95 % CI 1.7–109) and bakery workers (PR 2.62, 95 % CI 1.9–3.6).

The correlation between these two variables necessitated caution when offering both variables to the same model. Models where adjustment for atopy and specific sensitization was desired were first constructed separately and estimates were compared with those from models including both variables. In the end, estimates from the separate models were comparable and both variables were offered into all of the combined models.

In general, auto body shop workers tended to report more respiratory symptoms, while bakery workers tended to report more skin symptoms. This could be due, in part, to differences in exposure prevention activities. Unfortunately, self-reported use of personal protective equipment was only available for auto body shop workers, preventing a comparison of this effect. Observations by the researchers in the field suggest that differences did exist between the two populations, specifically that bakery workers did not use hand or respiratory protection while auto body shop workers tended to use both. A significant exposure–response relationship was observed in the auto body shop workers, the group observed to use PPE, suggesting that in these workers PPE use did not reduce exposure to a level that was trivial with respect to health effects.

Estimates of airborne exposure were used in the exposure–response models as a crude proxy for skin exposure, so results should be interpreted as airborne exposure-skin symptom associations. It is plausible that the airborne exposure estimates provide a good surrogate of skin exposure. Results from previous studies have shown a relatively strong association between skin and airborne exposures in auto body shop workers (Fent et al. 2008; Liljelind et al. 2010). No reports comparing skin and airborne exposures in bakery workers were located. It is possible that airborne exposure may be a better surrogate for skin exposure in the auto body shops, resulting in less exposure misclassification among auto body shop workers compared to bakery workers. It may also be that average isocyanate exposure (μg-NCO*m^−3^), or another exposure which was correlated with diisocyanates, was the causal exposure for skin symptoms in auto body shop workers, but that an exposure other than average wheat exposure (μg-wheat*m^−3^) was responsible for skin symptoms among bakery workers (i.e., wet work, oils, etc.).

Despite the observed associations between atopy, specific sensitization, and skin symptoms, the exposure–response relationships remained unchanged in sensitivity analyses. When atopic and specifically sensitized subjects were excluded from the models, the exposure–response relationships for skin symptoms in auto body shop workers persisted and the effect estimates were not attenuated. This provides support for the existence of an exposure–response relationship between NCO exposure and skin symptoms (work-related and non-work-related) in auto body shop workers.

In the second analysis, reported skin symptoms were predictive of reporting respiratory symptoms in both occupational groups regardless of the symptom combination, an association that has rarely been investigated (Lynde et al. 2009). Results were unchanged after adjustment for age, sex, smoking, and atopy. The persistence of the association after adjustment for these variables suggests that there are other factors that lead to the co-existing skin and respiratory symptoms (i.e., exposure). These results highlight the importance of considering both skin and respiratory outcomes in exposed workers as well as the importance of properly assessing both skin and airborne exposure in the workplace.

In conclusion, reporting skin symptoms was strongly and consistently associated with reporting respiratory symptoms in both bakery and auto body shop workers. Additionally, exposure–response relationships for skin symptoms were observed in auto body shop workers; similar relationships for work-related skin symptoms in bakery workers did not reach statistical significance. There are several reasons why an association may have been missed in bakery workers, including poor correlation between airborne and skin exposure for the particulate exposure and the lack of information on other, potentially causal, exposures in the workplace. The lack of observed association in bakery workers should be interpreted cautiously; exposure–response relationships for skin symptoms require more investigation in all occupations. These relationships must be better understood before more complex relationships are investigated; however, the overall goal remains the reduction of both airborne and skin exposure.

## Electronic supplementary material

Below is the link to the electronic supplementary material.

Supplementary material 1 (DOCX 174 kb)
