# Supplementary material for: Skin symptoms in bakery and auto body shop workers: associations with exposure and respiratory symptoms
Source: Int Arch Occup Environ Health. 2012 Mar 13;86(2):167–75. doi: 10.1007/s00420-012-0760-x (PMC3555349; doi:10.1007/s00420-012-0760-x)
Supplement: Supplementary file 1 — Supplementary material 1 (DOCX 174 kb) [file 420_2012_760_MOESM1_ESM.docx]

Supplementary Figure 1 Auto body shop workers associations between average isocyanate exposure and respiratory symptoms, shown in smoothed plots, stratified by atopy. Data rugs indicate the distribution of observations by exposure level. (a) Asthma-like symptoms in atopic subjects (linear: NS; spline: NS), (b) Work-related chest tightness in atopic subjects (linear: NS; spline: df=3, p<0.05), (c) Asthma-like symptoms in non-atopic subjects (linear: p<0.05; spline: NS), (d) Work-related chest tightness in nonatopic subjects (linear: p<0.05; spline: df=3, p<0.05).


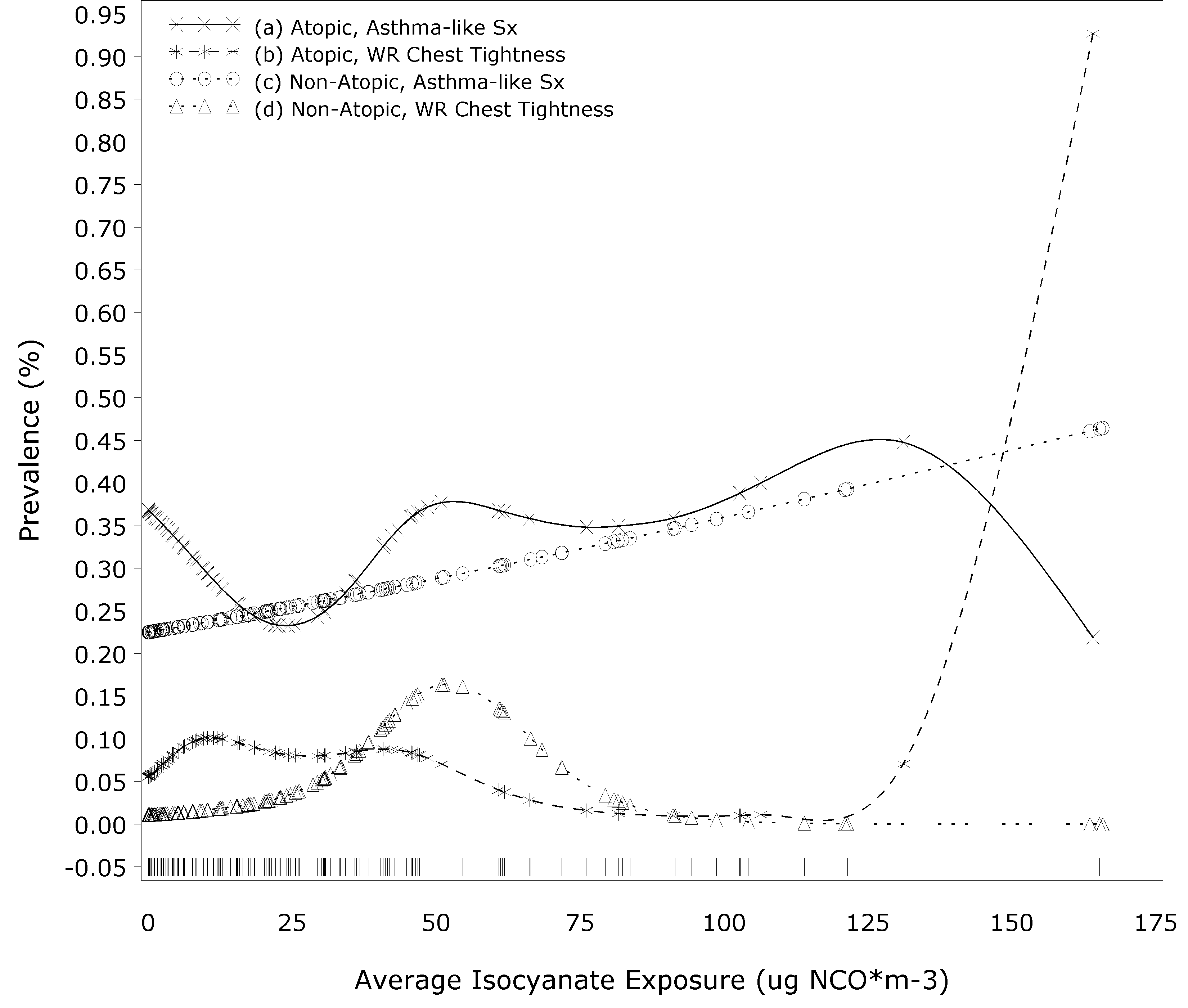


Supplementary Figure 2 Bakery workers associations between average wheat exposure and respiratory symptoms, shown in smoothed plots, stratified by atopy. Data rugs indicate the distribution of observations by exposure level. (a) Asthma-like symptoms in atopic subjects (linear: NS; spline: NS), (b) Work-related chest tightness in atopic subjects (linear: NS; spline: NS), (c) Asthma-like symptoms in non-atopic subjects (linear: NS; spline: NS), (d) Work-related chest tightness in non-atopic subjects (linear: NS; spline: NS).


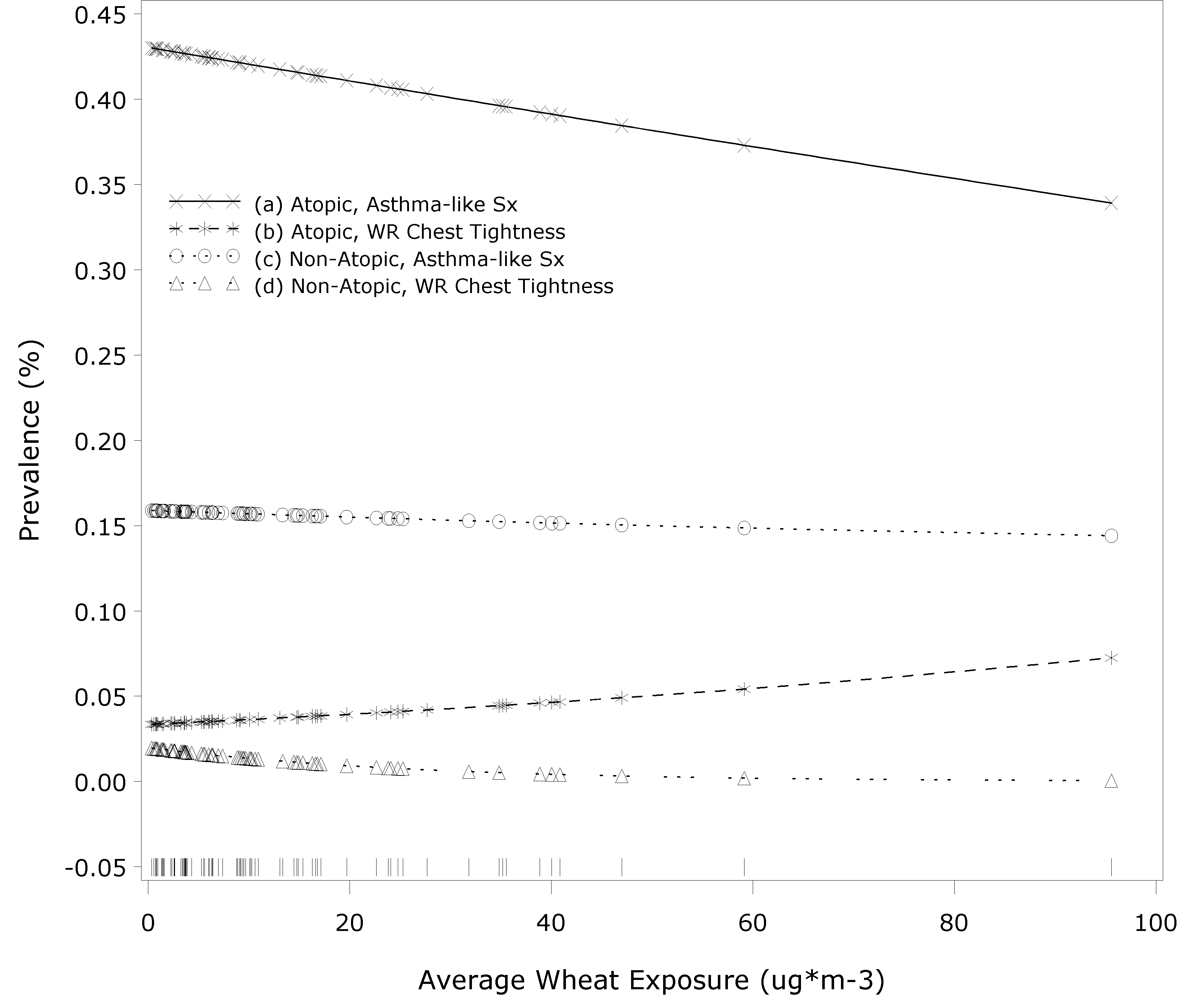


Supplementary Table 1 Results of generalized linear models describing the simple relationship between exposure, symptoms, atopy and specific IgE. Each reported prevalence ratio (PR) was estimated from a separate model. Models adjusted for age and sex. Symptom models additionally adjusted for smoking. (WR=Work-related)

|  | Independent Variable | Dependant Variable | PR (95% CI) |
| --- | --- | --- | --- |
| *Auto Body Repair Workers (n=473)* | |  |  |
|  |  |  |  |
|  | Average Isocyanate Exposure | Asthma-like Symptoms | 1.12 (0.9-1.4) |
|  | (ug-NCO*m^-3^) | WR Chest Tightness | 1.71 (0.8-3.5) |
|  |  | Atopy | 0.83 (0.7-1.0) |
|  |  | HDI-Specific IgE | 10.0 (1.6-72) |
|  |  |  |  |
|  | Atopy | Asthma-like Symptoms | 1.40 (1.1-1.9) |
|  |  | WR Chest Tightness | 2.61 (1.1-6.3) |
|  |  |  |  |
|  | HDI-Specific IgE | Asthma-like Symptoms | 1.13 (0.4-3.0) |
|  |  | WR Chest Tightness | 4.89 (1.3-18) |
|  |  |  |  |
| *Bakery Workers (n=723)* | |  |  |
|  |  |  |  |
|  | Average Wheat Exposure (ug*m^-3^) | Asthma-like Symptoms | 0.89 (0.7-1.1) |
|  |  | WR Chest Tightness | 0.92 (0.4-1.9) |
|  |  | Atopy | 0.91 (0.8-1.1) |
|  |  | Wheat-Specific IgE | 1.12 (0.8-1.5) |
|  |  |  |  |
|  | Atopy | Asthma-like Symptoms | 2.67 (2.1-3.4) |
|  |  | WR Chest Tightness | 4.11 (1.4-12) |
|  |  |  |  |
|  | Wheat -Specific IgE | Asthma-like Symptoms | 2.6 (2.0-3.4) |
|  |  | WR Chest Tightness | 15.7 (5.5-45) |
|  |  |  |  |
